# Performance Comparison of Spiral-Wound and Plate-and-Frame Forward Osmosis Membrane Module

**DOI:** 10.3390/membranes10110318

**Published:** 2020-10-30

**Authors:** Sungyun Lee

**Affiliations:** 1 Department of Civil Environmental Engineering, School of Disaster Prevention and Environmental Engineering, Kyungpook National University, 2559 Gyeongsang-daero, Sangju-si, Gyeongsangbuk-do 37224, Korea; sungyunlee@knu.ac.kr; 2 Department of Environmental Machinery, Korea Institute of Machinery and Materials, Daejeon 34103, Korea

**Keywords:** forward osmosis, spiral-wound, plate-and-frame, pressure drop, serial connection, footprint

## Abstract

We compared two representative forward osmosis (FO) modules—spiral-wound (SW) and plate-and-frame (PF)—to provide practical information for the selection of FO element for a large-scale FO process. The FO operating performance of commercially available SW FO and PF FO was explored under different membrane area and flow rate conditions. The performance trend as a function of the membrane was obtained by adjusting the number of serially connected elements. Although SW FO and PF FO elements exhibited comparable feed pressure drops, SW FO demonstrated a significantly higher draw channel pressure drop than PF FO. Furthermore, the significant draw pressure drop in SW FO increased the draw inlet pressure, consequently limiting the number of serially connected elements. For example, the maximum number of serially connected elements for the normal operation was three elements for SW FO (45.9 m^2^) but nine elements for PF FO (63 m^2^) when the flow rate of 10 LMP was applied for feed and draw streams. Additionally, a footprint analysis indicated that SW FO module exhibited a slightly larger footprint than PF FO. Under investigated conditions, PF FO exhibited relatively better performance than SW FO. Therefore, this pilot-scale FO study highlighted the need to reduce the flow resistance of SW FO draw channel to take advantage of the high packing density of the SW element.

## 1. Introduction

In the past decade, forward osmosis (FO) technology has gained much attention for various applications, such as desalination, wastewater reclamation, food concentration, and microalgae dewatering [1,2,3]. The main merit of the FO technology is that it utilizes osmotic pressure as the driving force for water transport; thus, the FO process needs lower energy than the pressure-driven membrane process, i.e., reverse osmosis (RO). Moreover, it has been reported that the FO membrane process has advantages in membrane maintenance due to its relatively loose and reversible fouling properties. However, to commercialize the FO process, there remain challenges, including the development of readily and completely separable draw solutes and improvement of high-performance FO membranes [4,5].

The osmotic pressure difference across a membrane mainly determines the water transport from a diluted solution (i.e., feed solution (FS)) to a concentrated solution (i.e., draw solution (DS)). To utilize the full osmotic pressure difference potential energy and obtain high membrane flux, the membrane should have intrinsic characteristics of high water permeability (*A*), low solute permeability (*B*), and low membrane structural parameter (*S*) [6]. Here, *B* involves the leakage of a draw solute into the FS, which can reduce the available osmotic pressure difference. Large *S* can induce significant dilutive internal concentration polarization inside the porous support layer due to restrained mass transfer, resulting in a considerable flux decline [7]. Thus, recent studies have been conducted on the fabrication of FO membranes to modify the characteristics of the selective layer and structure of the support layer using novel materials and additives [8]. 

These FO membranes are classified into two configurations as a flat sheet and hollow fiber membranes. However, hitherto, most commercial FO membranes have been developed in flat sheet form [9]. Membrane elements with flat sheet configurations are fabricated as spiral-wound (SW) or plate-and-frame (PF) types. As shown in Table 1, the SW element type is advantageous in terms of its relatively higher packing density (300–1000 m^2^/m^3^) and robustness during high-pressure applications [10]. Accordingly, the SW element type is commonly used for RO membranes. The SW FO and SW RO elements exhibit similar shapes so that the combination of FO and existing RO processes is considered easy. Due to the advantages of the SW element type, the SW FO element was commercialized earlier than the PF FO element, and most FO pilot studies have been conducted using SW FO elements. The first commercial SW FO element was produced by Hydration Technology Innovations (HTI) (Albany, OR, USA) around 2010 [11], and different commercial SW FO elements can now be obtained from Fluid Technology Solutions (Albany, OR, USA), Oasys Water (Boston, MA, USA), and Toray Advanced Materials Korea (Seoul, Korea) [1,12].

Unlike RO elements, FO elements are not employed at high pressure, and two solutions, including FS and DS, are introduced into the SW FO element. The FS flows through the mesh-spacers between membrane envelopes like RO elements, while the DS flows within the membrane envelopes corresponding to the permeate channel of RO elements. The conventional narrow permeate channel of RO elements is unsuitable for the DS flow into the membrane envelope from the central pipe. Thus, commercial SW FO elements adopt thick spacers for the channel inside the envelope to induce smooth draw flow [13]. However, some pilot studies have still reported high pressure drop in the draw channel of commercial SW FO elements [13,14,15,16]. Besides, the thick spacer of SW FO can reduce the packing density, which is the advantage of the SW element. 

Considering the PF element, the low packing density (100–400 m^2^/m^3^) is the major disadvantage due to the high flow channel height. However, the high channel allows the application of viscous liquid and wastewater with high fouling potential [19]. Furthermore, PF elements have been used in processes involving two different streams in the elements, such as electrodialysis, membrane distillation, and heat exchanges. Because the FO process also involves two streams, the PF element type can be suitable for the FO application. Currently, commercial PF FO elements are fabricated by Porifera [1,12]. 

These different features of SW FO and PF FO suggest the need to compare the FO performances for the selection of an appropriate element type. According to a mathematical model comparing the performance of SW FO and PF FO element [20], SW FO exhibited a higher flux than PF FO. However, it was noted that the water flux difference was not significant, and the water flux performance can be changed depending on the FO module design. Recently, a FO system with multiple SW FO elements is analyzed using MATLAB software (Mathworks, Inc., Natick, MA, USA) [21], but the FO modeling study did not investigate the effect of pressure drops on FO performance. Some pilot-scale FO studies have been conducted with SW FO elements, but there are very limited studies using PF FO elements. In addition, most pilot-scale FO researches have considered only a single element, leading to the inability to investigate operational issues in a large-scale system with serially connected elements. 

The objective of this study is to investigate the effect of different element types on FO performance. Therefore, we systematically evaluated the FO performance using commercially available SW FO and PF FO as a function of the membrane area and different operating conditions. The membrane area was adjusted by changing the number of elements connected in series, and we examined the pressure drops in feed and draw channels, water permeate, and FO performance efficiencies. Moreover, the footprints of SW FO and PF FO modules were compared to provide further information for membrane-type selection. The results of this study provide important design factors of FO elements for a large-scale FO process. 

## 2. Materials and Methods

### 2.1. FO Membrane Module

**Figure 1:**
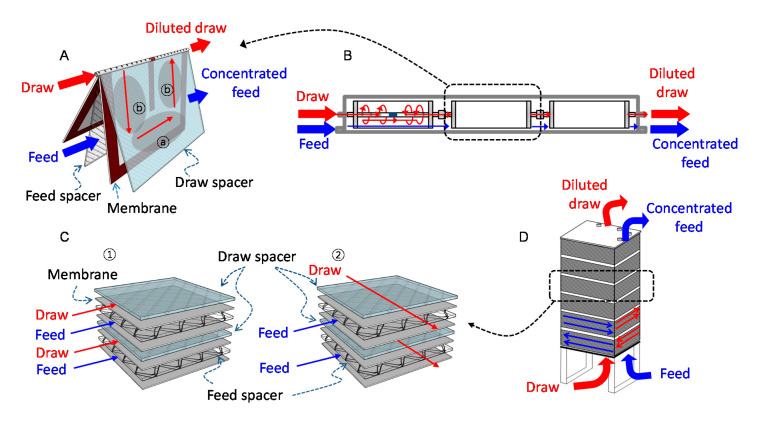
Schematic diagram of the spiral-wound (SW) and plate-and-frame (PF) forward osmoses (FO) module. (**A**) Flow directions of draw solution and feed solution in the SW FO element, (**B**) SW FO module comprising elements connected in series inside a vessel, (**C**) ①: Co-current flow in ⓐ the zone of SW FO element, ②: Cross-current flow in PF FO and ⓑ zone of SW FO elements, (**D**) PF FO module comprising stacked elements connected in series.

Two different types of commercial FO membrane elements—SW and PF FO membranes—were used in this study. Both SW FO (Toray Advanced Martials Korea Inc., Seoul, Korea) and PF FO (Porifera, Hayward, CA, USA) elements comprised polyamide thin-film composite (TFC)-based membranes. 8040 SW FO elements (8-inch diameter and 40-inch length) of the same size as the typically used RO elements were employed to investigate the SW FO performance. The element comprised 12 membrane envelopes, and the total effective membrane area was 15.3 m^2^. Each membrane envelope had a special spacer for the DS channel (1.98 mm), and an additional glue line at the center of the envelope was designed as a baffle for the DS flow path. Since the central pipe tube was blocked, the DS flew inside the membrane envelope and exited through the tube (Figure 1A). FS flew outside the membrane envelopes through the FS channel spacer (1.15 mm). Therefore, as depicted in Figure 1A,C, there were co-current and cross-current flow zones in the SW FO element. The elements can be serially connected using central pipe connectors in the membrane vessel (Figure 1C). 

The PF FO element comprised 33 cell-frames with a total effective membrane of 7 m^2^. The element had a fishnet-type spacer (1.15 mm) in the feed channel, but the draw channel was spaced with many protrusions on the plate surface without an additional draw spacer. In an element, the cell frames were internally connected in parallel, and the FS and DS flew in the cross-current flow direction, as shown in Figure 1C②. Each PF FO element can be serially connected via the stacking configuration of the elements (Figure 1D). 

### 2.2. Experimental Setup

Two pilot-scale FO systems were adopted for investigating SW FO and PF FO membranes. The schematic diagram and details about the FO systems are provided in our previous study [15,22]. Briefly, the FO systems were equipped with magnetic flow meters, digital pressure gauges, and conductivity meters at the inlet and outlet of FS and DS sides, and the online data were recorded using a connected computer. The feed flow rates were changed to 10, 15, and 20 LPM, while the draw flow rates were applied at 5 and 10 LPM. Both FS and DS were prepared using NaCl and tap water (19.5 ± 1 °C). The concentrations of FS and DS were 10 g/L and 70 g/L, respectively. To investigate the impact of the membrane area on the FO performance, three SW FO modules, including one SW FO element per vessel, were equipped, and control valves and pipes were installed for serial-connection simulation. For PF FO, three different PF FO modules with one, three, and six elements used and the two modules with three and six elements were serially connected (total nine FO elements) by the controlling valves [22]. In both FO systems, the feed pressures were carefully controlled using back-pressure valves to keep the feed side pressure similar to or higher than the draw side pressure to avoid membrane damage by a negative transmembrane pressure. 

The membrane characteristics, including water permeability (*A*), sodium chloride permeability (*B*), and membrane structural parameter (*S*), were analyzed using coupon-sized FO membranes in a bench-scale experiment, as reported in our previous study [23]. 

### 2.3. Analysis of FO Membrane Performance

The FO performance of SW FO and PF FO modules was evaluated in terms of pressure drop, water permeate, average FO membrane flux, feed volume reduction, and performance efficiency. The impact of the membrane area on the FO performance was examined by changing the number of FO elements connected in series. Moreover, the effect of feed and draw flow rates on the FO performance was also tested by changing the feed flow rates within the range of 10–20 LPM at a constant draw flow rate of 5 or 10 LPM. 

The average FO membrane fluxes (*J_w_*, L m^−2^ h^−1^) were obtained using water permeate (in LPM), which was obtained from feed flow rate difference divided by total effective membrane area, as follows:

(1) $$J_{w}=\;\frac{Q_{feed,\;in}-Q_{feed,\;out}}{A_{m}}$$

where *A_m_* is the total effective membrane area (m^2^), *Q_feed,in_* and *Q_feed,out_* are the feed flow rate (LPM) at the inlet and outlet of the module, respectively. The feed flow decreased through the membranes because the feed water permeated to the draw side in the FO module. The feed volume reduction was expressed as normalized feed volume at the outlet as follows:

(2) $$\mathrm{normalized}\;\mathrm{feed}\;\mathrm{volume}\;\mathrm{at}\;\mathrm{outlet}=\;\frac{Q_{feed,\;out}}{Q_{feed,\;in}}$$

Feed water recovery (*R*) and normalized feed volume at the outlet had the following relationship:

(3) $$R=\frac{Q_{feed,\;in}-Q_{feed,\;out}}{Q_{feed,\;in}}\;=1-\mathrm{normalized}\;\mathrm{feed}\;\mathrm{volume}\;\mathrm{at}\;\mathrm{outlet}$$

In FO modules, the feed water transported across the membranes until the concentrations of FS and DS came to equilibrium. Therefore, the theoretical maximum water recovery, *R_max_*, can be calculated using the mass balance of FS and DS, as well as the membrane characteristics. The *R_max_* values were calculated using the following formula, as described in the literature, in a co-current flow of FS and DS [22,24]

(4) $$R_{max}=\frac{\left(1-\phi \right)\left(C_{draw,\;in}-C_{feed,\;in}\right)}{\phi C_{feed,\;in}+\left(1-\phi \right)C_{draw,\;in}+\left(B/vAR_{g}T\right)}$$

where $C_{feed,\;in}$ and $C_{draw,\;in}$ are the inlet molar concentrations of FS and DS, respectively; v the van’t Hoff dissociation factor, $R_{g}$ the universal gas constant, and T the absolute temperature. The feed flow rate fraction (ϕ) was defined in terms of both the initial feed ($Q_{feed,\;in}$) and draw flow rate ($Q_{draw,\;in}$) as follows:

(5) $$\phi =\;\frac{Q_{feed,\;in}}{Q_{feed,\;in}+Q_{draw,\;in}}$$

Using the *R_max_* value, the theoretical minimum normalized feed volume (theoretical *Q_feed, out_*/*Q_feed, in_)* can also be calculated using Equations (2) and (3). 

Consequently, the FO performance efficiency was obtained using the ratio of experimentally obtained feed water recovery (*R_exp_*) and theoretical maximum water recovery (*R_max_*).

(6) $$\mathrm{FO}\;\mathrm{performance}\;\mathrm{efficiency}=\;\frac{R_{exp}}{R_{max}}$$

The driving force of water flux in FO is the osmotic pressure difference between DS and FS. The effective concentrations of DS and FS were influenced by the hydrodynamic conditions of the channels and the membrane properties. Considering the internal and external concentrations of the membrane, the water flux in FO can be written according to the following equation [25]:

(7) $$J_{w}=A\left[\frac{\pi_{D,b}\exp\left(-\frac{J_{w}S}{D}\right)-\pi_{F,b}\exp\left(\frac{J_{w}}{k}\right)}{1+\frac{B}{J_{w}}\;\left[\exp\left(\frac{J_{w}}{k}\right)-\exp\left(-\frac{J_{w}S}{D}\right)\right]}\right]$$

where *π_D,b_* and *π_F,b_* are the bulk osmotic pressures of DS and FS, and *D* the diffusion coefficient of NaCl. The mass transfer coefficient, *k*, can be calculated from the geometry and experimental conditions [26,27,28], and 1.46 × 10^−4^ m s^−1^ was used to estimate water flux of the investigated membranes as a function of feed and draw concentrations.

## 3. Results and Discussion

### 3.1. Characteristics of SW FO and PF FO Elements 

In this study, the effect of FO membrane element types on FO performance was investigated. The best comparison of the element types can be achieved using different elements comprising the same FO membrane. However, such FO elements are not commercially available; therefore, we conducted the FO tests using two representative FO elements—SW FO and PF FO—with similar TFC polyamide-based membranes. 

Table 2 shows the membrane characteristics of SW FO and PF FO. The water permeability value of SW FO was 6.68 L m^−2^ h^−1^ bar^−1^, which was approximately three times higher than that of PF FO. Based on the water permeability value, it could be expected that the SW FO membrane would provide much higher FO water flux than the PF FO. However, when 1 M NaCl and deionized water were used as DS and FS, respectively, the FO flux of SW FO was 34.2 L m^−2^ h^−1^, which was only 32% higher than that of PF FO. The difference in FO flux seemed relatively small compared with the difference in water permeability. Since the FO flux was determined by *A*, *B*, and *S* values of the membranes, as described in Equation (7), this result could be interpreted based on the *B* and *S* values of the membranes. For high FO water flux, the FO membrane should have not only high *A* but also low *B* and small *S* values [29,30]. However, the NaCl permeability value (0.54) of the SW FO was higher than that (0.49) of PF FO membranes, which could induce higher reverse solute flux. Moreover, SW FO had a higher *S* value (336 μm) than PF FO (269 μm), which led to higher internal concentration polarization in the SW FO membrane, consequently reducing the water flux.

**Figure 2:**
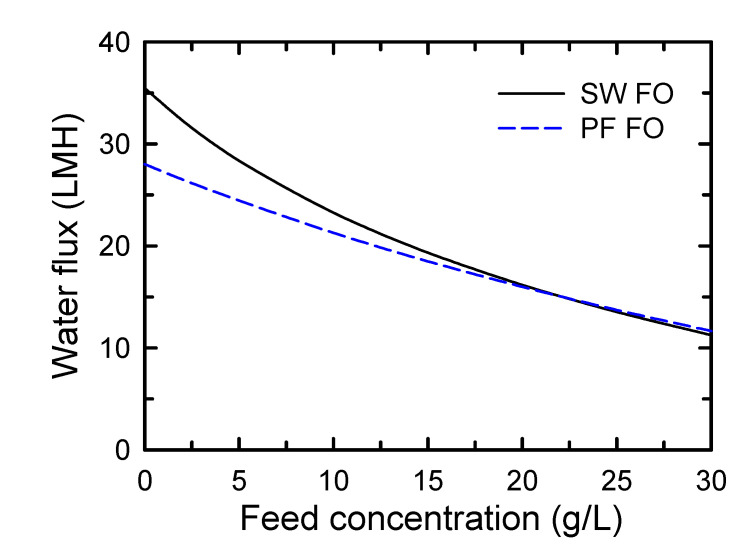
Water flux estimation of spiral-wound and plate-and-frame forward osmoses membranes as a function of feed solution concentration based on *A, B*, and *S* values in Table 2 and Equation (7). A 70 g/L of draw solution concentration was employed for the estimation.

During the pilot-scale test in this study, the difference in FO fluxes was predicted to decrease when applying the same concentrations of DS and FS. Figure 2 shows the estimation of the FO membranes as a function of feed concentration based on the *A*, *B*, and *S* values of the membranes using Equation (7). For the simulation, the applied DS concentration was fixed at 70 g/L, which was the same concentration for our pilot-scale experiments. According to the estimation, the FO flux of SW FO and PF FO was 23.3 and 21.3 LMH (L m^−2^ h^−1^), respectively, at a feed concentration of 10 g/L. This slightly higher FO flux of the SW FO membrane was estimated to be closer to that of PF FO as FS concentration increased. Since the feed concentration would increase with the flow through the membranes in the module, it was expected that the FO flux of SW FO and PF FO would be similar in the module-scale FO system. Despite having a high *A* value, the severe water flux reduction of SW FO was considered to be due to the combined effect of external concentration polarization of FS high concentration, and the driving force reduction across the membrane was due to internal concentration polarization [32] and low solute selectively. Additionally, it seemed that the solute selectively became more significant at FS concentration greater than 21.8 g/L, resulting in a higher flux of PF FO than the SW FO membrane (Figure 2). 

Based on Figure 2, it is considered that SW FO elements would provide similar or somewhat higher FO flux than PF FO elements until the feed flow decreased to approximately 50% of initial feed flow by FO elements. The experimental results related to FO flux and operational pressures in the FO modules are discussed in the following sections. 

### 3.2. Performance of a FO Element 

For large-scale applications, FO membranes are applied as a unit of an element or a vessel containing multiple FO elements. In this section, the performance of SW FO and PF FO elements was compared in terms of pressure drops, water permeates, feed volume reduction, and average flux. 

**Figure 3:**
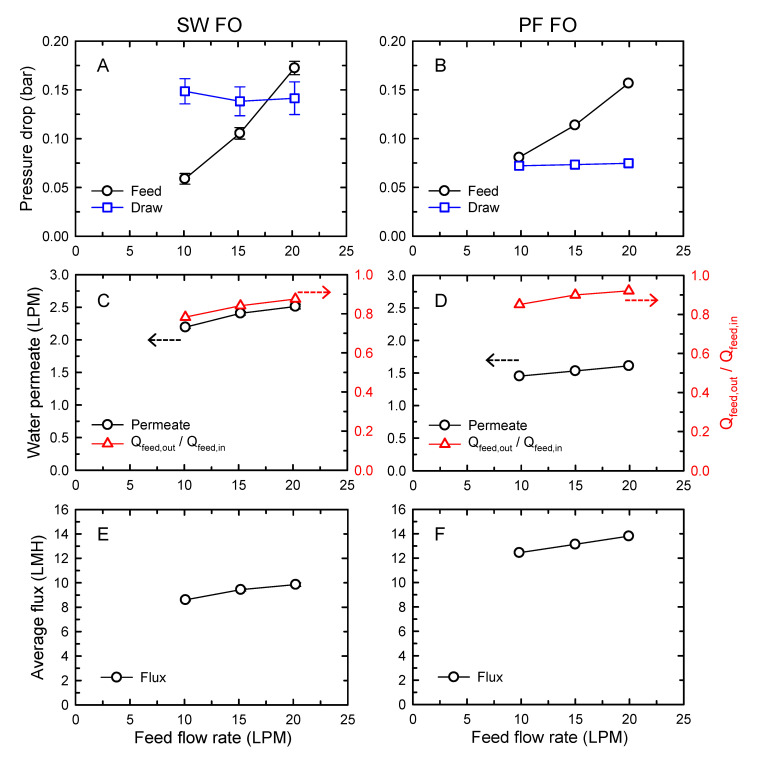
Performance of a forward osmosis (FO) element as a function of feed flow rates: spiral-wound (SW) FO (**A**,**C**,**E**) and plate-and-frame (PF) FO (**B**,**D**,**F**) elements. Pressure drops in feed and draw channels (top), the water permeate and normalized feed flow volume (*Q_feed, out_*/*Q_feed, in_*) at the outlet (middle), and the average flux (bottom). The draw flow rate was fixed at 5 LPM. The concentrations of feed and draw solutions were 10 and 70 g/L, respectively.

Figure 3 shows the performance of SW FO and PF FO as a function of FS flow rates with constant DS flow at 5 LPM. It should be noted that the membrane area in an element of SW FO and PF FO was 15.3 and 7 m^2^, respectively. In Figure 3A,B, the pressure drops in feed and draw channels represented the channel resistance, which could influence the operating pressure. The pressure drops in the feed channel of SW FO and PF FO increased as the feed flow rates increased in the range of 10–20 LPM. Although the SW FO had more than twice the membrane area than PF FO, the pressure drops in the feed channel of the elements were comparable. Relatively small flow resistance in the feed channel of SW FO was likely due to higher feed spacer thickness (Table 2). However, it was observed that SW FO had a slightly higher increment rate of feed pressure drop, rising from 0.06 to 0.17 bar. As described below, this higher increment rate was considered to be related to the high pressure drops in the draw channel of SW FO and the operation method of the FO system. 

The pressure drop in the draw channel was 0.14 bar for the SW FO element, which was higher than 0.07 bar for the PF FO element, and the pressure drops of both FO elements did not change significantly regardless of the feed flow rate change. Specifically, for PF FO, the drops in the draw pressure were lower than those in the feed pressure under all the investigated feed flow rates, while SW FO exhibited a higher drop in the draw pressure than that of the feed pressure when low feed flow rates were employed (feed flow rates of 10 and 15 LPM). Since the operating pressure of the feed side should be higher than that of the draw side to avoid membrane damage, this higher pressure loss in the draw channel of SW FO required an increase of inlet feed pressure with control of the back-pressure valve. Consequently, it induced the higher increment rate of feed pressure drop for SW FO with increasing feed flow rates. This result implied that the overall operating pressure of the SW FO system would be high, which could limit the number of the elements in series connection. 

The water permeate and the normalized feed volume at the element outlet as a function of FS flow rates are presented in Figure 3C,D. Here, the lower normalized feed volume indicated the higher volume reduction by the FO system. The FO water permeates of both SW FO and PF FO elements increased with increasing applied feed flow rates, from 2.19 to 2.51 LPM for SW FO and from 1.45 to 1.61 LPM for PF FO. Despite the increase in water permeates, the normalized feed volume at the outlet increased with increasing feed flow rates. This observation corresponded with previous findings, which slightly improved water flux by feed flow increase and did not result in higher water recovery due to a relatively high feed flow rate compared to the permeate improvement [22,33].

The lowest normalized feed flow volume at the membrane outlet was observed as 0.78 when 10 LPM of feed flow rate was applied to SW FO. Considering the feed volume reduction ratio of 0.78, the FS concentration was expected to increase from 10 to 12.8 g/L. Meanwhile, DS concentration would be decreased from 70 to 48.6 g/L, according to the mass balance. Since there was still a concentration difference between FS and DS, the lower normalized feed volume at the outlet could be achieved using serial connection with additional FO elements.

The higher permeate and feed volume reduction by SW FO would be mainly due to the larger membrane area per element. Although slightly higher water flux of SW FO was estimated in Figure 2, the effect of the difference in the membrane area resulted in opposite behavior of the average water flux of SW FO and PF, as depicted in Figure 3E,F. The water flux of SW FO and PF FO increased from 8.60 to 9.91 LMH and 12.45 to 13.80 LMH, respectively. The performance of the permeate and the membrane flux of SW FO and PF FO in terms of membrane area is detailed in the following section.

### 3.3. Performance of Serially Connected FO Elements

**Figure 4:**
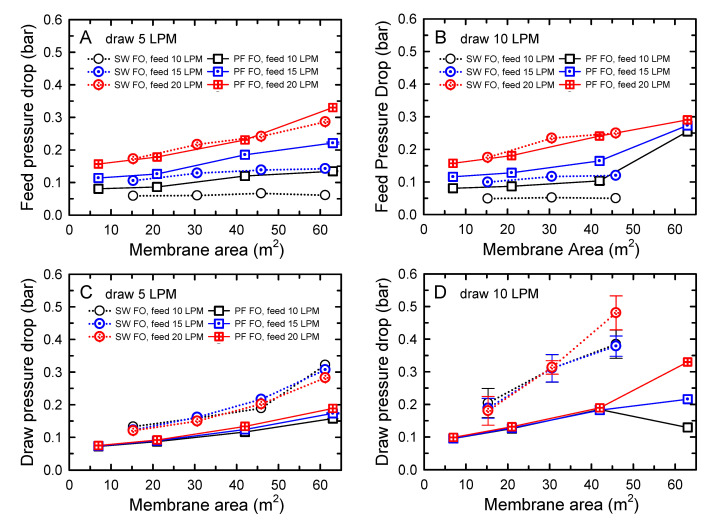
Comparisons of pressure drops in the feed (**A**,**B**) and draw (**C**,**D**) channels of spiral-wound (SW) and plate-and-frame (PF) forward osmoses (FO) as a function of membrane area. The applied draw solution (DS) flow rates were 5 (**A**,**C**) and 10 LPM (**B**,**D**). The feed solution (FS) flow rates were varied from 10 to 20 LPM. The membrane areas were adjusted by a serial connection of FO elements: 1–4 elements of SW FO and 1, 3, 6, and 9 elements of PF FO. The concentrations of FS and DS were 10 and 70 g/L, respectively.

The effects of membrane area on the performance of SW FO and PF FO were examined using different numbers of elements connected in series. Although SW FO and PF FO membranes comprised different areas per element, the performance trends were obtained using the graphs of SW FO and PF FO as a function of membrane area. The pressure drops in the feed and draw channels of SW FO and PF FO as a function of membrane area were compared and are presented in Figure 4. To investigate the effect of feed and draw flow rates on the pressure drops, two different draw flow rates of 5 and 10 LPM were applied with three different flow rates at 10, 15, and 20 LPM.

For the feed pressure drop analysis (Figure 4A,B), the drops in the feed pressure of SW FO elements were comparable or slightly lower than those of PF FO elements under the investigated flow rates and membrane area conditions. For example, when the feed flow rate of 10 LPM was applied, the feed pressure drops were within the range of 0.059–0.061 bar and 0.081–0.135 bar for SW FO and PF FO, respectively. Interestingly, the feed pressure drop values were similar regardless of the employed draw flow rates, indicating less effect on the membrane deformation due to the hydraulic pressure difference in the module suggested in previous studies [34,35].

Examining the effect of membrane area, minute increments in the feed pressure drops were observed with increasing membrane area and especially the negligible increment for both elements when low feed flow rates of 10 and 15 LPM were applied. Contrarily, the increased feed flow rates yielded a much greater increase in feed pressure drops. According to the Darcy equation, the hydraulic pressure drop in the channels can be expressed as a function of flow velocity and channel dimension [27]: ∆P_loss_ = *f* × *l*/*d_h_* × (*ρu*^2^)/2. Here, *f* is the frictional factor, *l* the length of the flow channel, *d_h_* the hydraulic diameter of the flow channel, *ρ* the fluid density, and *u* the fluid cross-flow velocity. Therefore, it can be concluded that the increase in flow velocity due to increased feed flow rate influenced the feed pressure drops more critically than the channel dimension factors, such as extended feed channel lengths by serial connection. When the feed flow rate of 20 LPM was applied, the effect of the membrane area was significantly observed. This result was likely related to the operation method associated with draw side pressure, as described in Section 3.2.

However, as depicted in Figure 4C,D, different trends were distinctly observed in the draw pressure drops of SW FO and PF FO. The SW FO possessed much higher draw pressure drops than PF FO under the tested conditions, as predicted by the result using one element (Figure 3A,B). This higher pressure drops of SW FO than PF FO could be attributed to the friction loss induced by the combination of longer draw channel length and characteristics of the channel design. Especially for the draw pressure drop in SW FO with 10 LPM of draw flow, a significant increase was observed with an increase in the membrane area. For example, when the membrane area of SW FO increased using up to three elements in series connection, the draw pressure drop increased from 0.20 to 0.38 bar and 0.13 to 0.19 bar with 10 and 5 LPM of draw flow, respectively. However, the variation of feed flow rate had little effect on the draw pressure drops, indicating the independence of each channel, which was similar to the observation in the feed channels.

**Figure A1:**
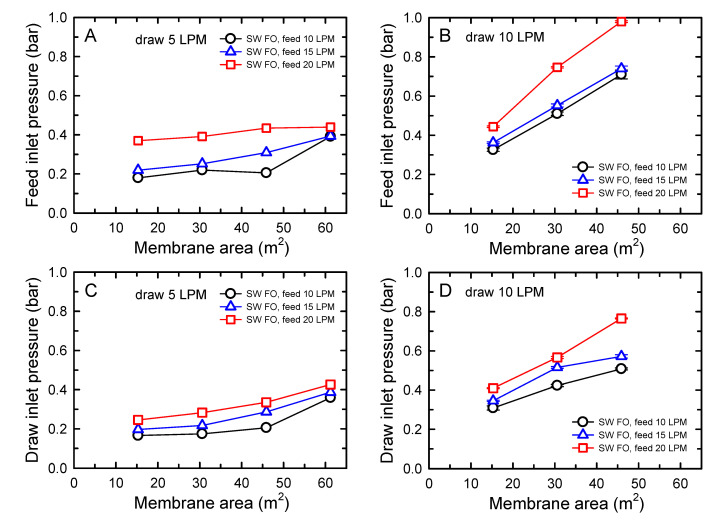
Inlet pressures in feed and draw channels of spiral-wound (SW) forward osmosis (FO) as a function of membrane area. The applied direct solution (DS) flow rates were 5 (**A**,**C**) and 10 LPM (**B**,**D**). The feed solution (FS) flow rates were fixed at 10 LPM. The membrane areas were adjusted via 1–4 elements serial connection of SW FO. The concentrations of FS and DS were 10 and 70 g/L, respectively.

**Figure A2:**
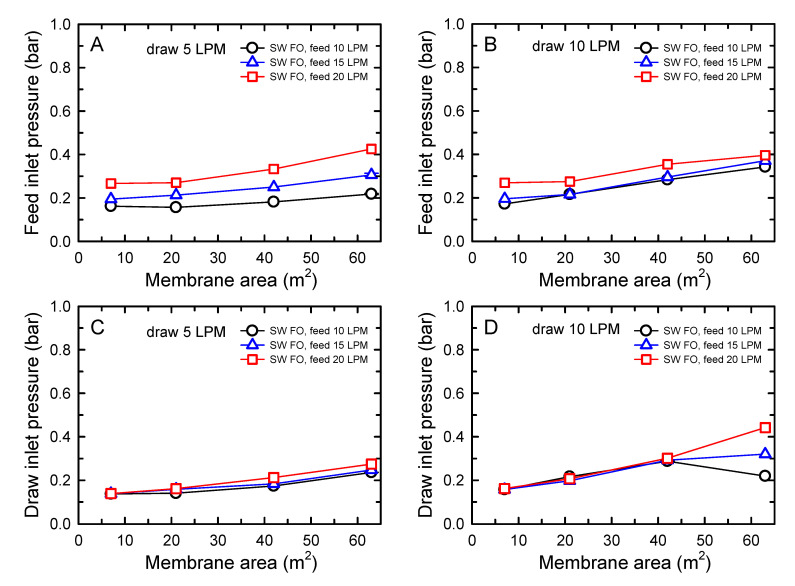
Inlet pressures in feed and draw channels of plate-and-frame (PF) forward osmosis (FO) as a function of membrane area. The applied direct solution (DS) flow rates were 5 (**A**,**C**) and 10 LPM (**B**,**D**). The feed solution (FS) flow rates were fixed at 10 LPM. The membrane areas were adjusted via serial connection of 1, 3, 6, and 9 elements of PF FO. The concentrations of FS and DS were 10 and 70 g/L, respectively.

A significant problem to be noted regarding the high pressure on the DS side of SW FO was that the high-pressure drop on the DS side caused an increase in its inlet operating pressure, which, in turn, led to an increase in the operating pressure of the FS side to prevent the membrane damage. This problem was very pronounced when 10 LPM of draw flow was applied, as shown in Figure A1B,D (Appendix A, Figure A1). Consequently, the increased operating pressure hindered the stable operation of the SW FO system with four elements connected in series at 10 LPM draw flow rate. Thus, the data points of SW FO in Figure 4B,D using four elements could not be obtained. With similar effects, PF FO also showed unstable draw pressure drops when nine elements were connected at a 10 LPM draw flow rate (Appendix A, Figure A2).

**Figure 5:**
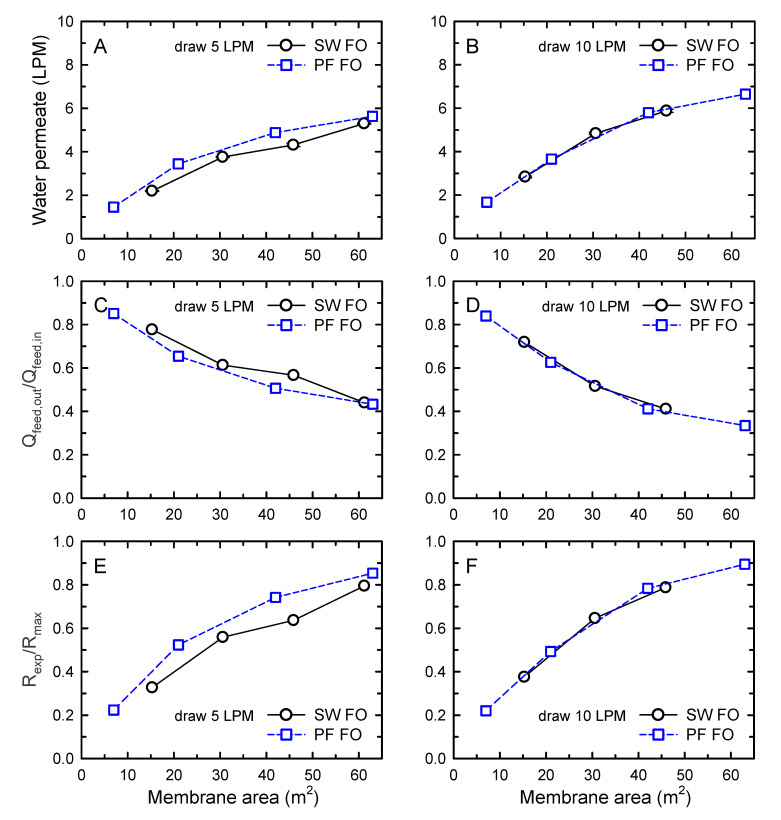
Comparison of FO performance of spiral-wound (SW) and plate-and-frame (PF) forward osmoses (FO) modules. The applied draw solution (DS) flow rates were 5 (**A**,**C**,**E**) and 10 LPM (**B**,**D**,**F**). The feed solution (FS) flow rates were fixed at 10 LPM. Water permeate (top), normalized feed flow volume (*Q_feed, out_*/*Q_feed, in_*) at the outlet (middle), and performance efficiency (*R_exp_*/*R_max_*) (bottom) as a function of the membrane area. The concentrations of FS and DS were 10 and 70 g/L, respectively.

In Figure 5 and Table 3, the performance of SW FO and PF FO elements was compared in terms of water permeates, feed volume reduction, and FO performance efficiency. Specifically, in Figure 5, the effects of different draw flow rates of 5 and 10 LPM on the performance of the two elements were depicted as a function of the membrane area with a constant feed flow rate of 10 LPM. Although from Figure 3, the absolute performance comparison of SW FO and PF FO was difficult when one element was used, the relative performance comparison of the elements was possible using the performance trends as a function of membrane area, as shown in Figure 5. According to Figure 2, the water permeate of SW FO was projected to be comparable or slightly higher than PF FO when the same membrane area was applied. Interestingly, the performance of SW FO and PF FO was observed to be similar to the predicted value, as presented in Figure 5. When 5 LPM draw flow rate was applied, the SW FO exhibited slightly decreased performance (Figure 5A,C,D), but the performance was improved to a value comparable to that of PF FO when a draw flow of 10 LPM was employed. Although the performance of both FO elements was improved under higher draw conditions, the reason the effect was more pronounced in SW FO could be attributed to the draw channel design of SW FO. As shown in Figure 1A, the configuration of the draw channel for SW FO seemed to limit the ability to induce smooth flow inside the membrane envelopes [36]. Thus, at low draw flow rates, mixing and flow would be more difficult within the draw channel, which was more likely to cause dead zones of draw flow. This result suggested the trade-off relation in SW FO operation between higher performance and lower operating pressure.

Table 3 summarizes the performance of SW FO and PF FO with the membrane areas of 45.9 and 42 m^2^, respectively, which were conducted at different feed and draw flow rates. Overall, the lower normalized feed flow volume at the membrane outlet was obtained with a lower initial feed flow rate. Under the tested conditions, the experimentally obtained normalized feed volume at the outlet was higher than the theoretical normalized feed volume, indicating that the membrane area was insufficient to utilize the full potential of the osmotic pressure difference between FS and DS. As shown in Figure 5F, FO performance increased with increasing membrane area, but in the actual FO process application, the FO system scale should be determined after considering the degree of the performance improvement, and the capital cost increased with an increase in the membrane area [5].

When the DS flow increased from 5 to 10 LPM, the FO performance efficiency of both elements was improved. For example, the performance efficiency of SW FO improved from 0.64 to 0.79 while that of PF FO improved from 0.74 to 0.78. The less performance difference of PF FO also could be related to the relatively smooth draw flow within the channel, as described earlier in the draw pressure drop results.

### 3.4. FO Footprint and Insights for FO Elements Design

**Figure 6:**
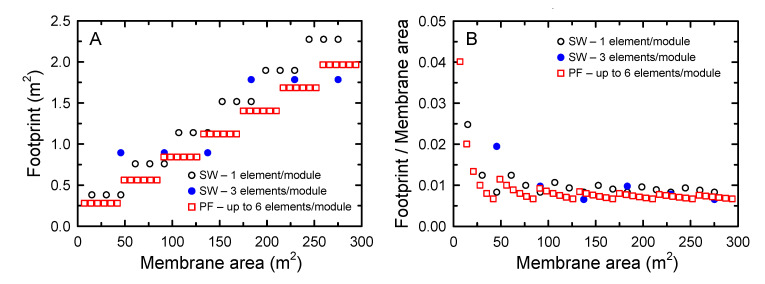
Estimation of the footprint (**A**) and footprint/membrane area (**B**) for SW FO and PF FO.

Civil engineering and membrane module in the RO desalination system account for 17.3% and 5.2% of total expenditure, respectively. RO membrane replacement accounts for 5.3% of operating expenditure [5]. Considering the low water permeate of FO compared to RO, the selection of an appropriate FO module with a lower footprint and higher water permeate performance is critical to avoid a large cost of the FO treatment process. The FO footprint was calculated as a function of membrane area, as presented in Figure 6, to compare the effect of elements types on the footprint. For SW FO, two different modules, which were one element per one vessel and three elements per one vessel, were projected. The maximum number of modules in the vertical direction was assumed to be three for SW FO, considering its installation and maintenance. For PF FO, the maximum number of stacked elements in serial connection was assumed to be six. The maximum number of serially connected elements for SW FO and PF FO were determined based on the number of elements that demonstrated stable performance, as shown in serial-connection experiments in Section 3.3.

Although the packing density varied depending on the elements’ design and configuration of the spacers, generally, SW type elements exhibited higher packing density than PF type elements. For example, the packing density of SW RO ranges from 300–1000 m^2^/m^3^, while that of PF elements ranges from 100–400 m^2^/m^3^ [18] (Table 1). For the investigated elements, SW FO and PF FO exhibited packing densities of 464 and 283 m^2^/m^3^, respectively (Table 2). Given the approximately 1.6 times higher packing density of SW FO, a much lower footprint of SW FO than that of PF FO was expected. To estimate the footprint of the SW FO and PF FO, the module dimensions, i.e., the vessels and supporting plates’ dimensions of SW FO and PF FO, respectively, were used. Here, the typical SW vessels dimensions of 0.25 × 1.5 m and 0.25 × 3.5 m (diameter × length) were used for one and three SW FO elements [29], respectively. The dimensions of the supporting plates for PF elements were 0.4 × 5.3 m [31], and the areas for connecting pipes and facilities were not considered for a rough analysis.

Contrary to the expectation based on the packing density, overall, the PF FO module exhibited a slightly lower footprint than SW FO modules (Figure 6). The footprint normalized by the membrane area ranged from 0.0099–0.0083, 0.0097–0.0065, and 0.0077–0.0067 m^2^/m^2^ for the SW FO module with one element, three elements, and PF FO module, respectively. The high footprint of SW FO could be mainly due to the limit of elements that could be connected in series. Although 6–8 SW RO elements were installed in a pressure vessel [37], only three elements of SW FO could be connected in series due to high flow resistance in the draw channel.

Based on the FO performance results of this study, the strategy for SW FO element design modification to reduce the draw pressure drop and footprint can be suggested as follows. The effect of introducing the DS in a parallel configuration can be improved with an increase in the number of membrane envelopes and a decrease in the width of the envelope. Accordingly, the effect of reduced flow length and draw flow velocity in the channel will lead to a reduction of flow resistance in the draw channel. Furthermore, the adoption of special central pipes for multiple SW elements connection, which allows DS flow through each parallel element, can mitigate the pressure drops in the draw channel. Examples of such central pipes have been characterized by passing DS parallelly to other SW elements, providing additional pipes in the central pipe [38], or providing a barrier in the longitudinal direction of the central pipe [39]. The SW FO with the lower draw pressure drops will enable an increase in the number of elements that can be connected in series, thereby reducing the footprint. Besides, the reduced draw pressure drops will mitigate the unwanted operating pressure build-up and reduce the energy consumption of the pump for the FO system [40].

## 4. Conclusions

In this study, we compared the FO performance of two element types—SW FO and PF FO—using multiple elements connected in series. The FO performance was investigated in terms of the pressure drops in feed and draw channels, water permeate, feed volume reduction, FO performance efficiencies, and footprint. Although the membrane area per element for SW FO and PF FO was different, the direct performance comparison was possible using the pronounced performance trends as a function of membrane area by controlling the number of elements connected in series. The results revealed that SW FO exhibited a higher pressure drop in the draw channel than PF FO. The high draw pressure drop of SW FO induced high inlet pressures of both feed and draw streams. Consequently, the increased operating pressure limited the number of elements in series connection. The significant impact of the draw flow rates on SW FO indicated that the increase in the flow velocity due to increased feed flow rate was a critical factor for high pressure drops. Considering the less number of membrane envelopes of SW FO compared to the number of cell-frames of PF FO and its effect on the draw flow velocity, we suggested the strategy of increasing the number of membrane envelopes and decreasing the width of the envelope for SW FO element design modification, which could mitigate the high pressure drops in the draw channel of SW FO. Interestingly, when sufficient FS and DS flow rates were applied, both elements exhibited similar performance in terms of water permeate and feed volume reduction, as projected from the model using the membrane characteristics of *A*, *B*, and *S*. Although it was expected that the footprint of SW FO would be less than that of PF FO due to higher packing density of SW FO, our footprint analysis revealed that PF FO module exhibited slightly less or similar footprint compared to SW FO modules. The footprint analysis asserted that to take advantage of the high packing density of SW FO, the reduction of the flow resistance of the draw channel was necessary to increase the number of elements for serial connection. Overall, the tested PF FO element provided relatively better performance than the SW FO element.

## Figures and Tables

**Table 1:** Typical characteristics for spiral-wound and plate-and-frame membrane modules and the commercial forward osmosis (FO) membrane manufacturers [17,18].

Parameter	Spiral-Wound	Plate-and-Frame
Feed flow channel height (mm)	0.7–0.9	1–3
Element size	diameter: 8, 4, or 2.5 inch, length: 40 inch	various
Packing density (m^2^/m^3^)	300–1000	100–400
Membrane area (m^2^/element)	37–41 (8-inch diameter element)	various
Advantage	high packing density, cost-effective construction	suitable for viscous liquid
Disadvantage	high pressure drop (100–150 kPa for an RO element), long permeate path, difficult to clean	low packing density
Typical application process	reverse osmosis, nanofiltration	membrane distillation, membrane bioreactor, electrodialysis
Commercial FO manufacturer	Toray Advanced Materials Korea Oasys Water Fluid Technology Solutions	Porifera

**Table 2:** The specifications of the spiral-wound (SW) and plate-and-frame (PF) forward osmoses (FO) membrane modules employed in this study.

Parameter	SW FO	PF FO
Water permeability, *A* (L m^−2^ h^−1^ bar^−1^) ^a^	6.68	2.22
NaCl permeability, *B* (L m^−2^ h^−1^) ^a^	0.54	0.49
Structural parameter, *S* (μm) ^b^	336	269
NaCl rejection, *R* (%) ^a^	97.4	96.0
FO water flux, *J_w_* (L m^−2^ h^−1^) ^b^	34.2	25.9
Feed spacer thickness (mm)	1.15	0.76 ^c^
Draw spacer thickness (mm)	1.98	none ^c^
Membrane area (m^2^/element)	15	7 ^c^
Packing density (m^2^/m^3^)	464	283

^a^*A*, *B*, and *R* values were obtained in a lab-scale RO mode test using NaCl 1000 mg/L at 3 bar. ^b^ *S* and *J_w_* were measured in a lab-scale FO mode test using 1 M NaCl and deionized water as draw and feed solutions, respectively. ^c^ Data was obtained from the manufacturer [31].

**Table 3:** Summary of FO performance for spiral-wound (SW) and plate-and-frame (PF) forward osmoses (FO) modules under different feed solution (FS) and draw solution (DS) flow rates conditions. Experimental normalized feed flow volume at outlet: *Q_feed, out_*/*Q_feed, in_*; theoretical minimum normalized feed flow volume at outlet: theoretical *Q_feed, out_*/*Q_feed, in_*; FO performance efficiency: *R_exp_*/*R_max_*. Three SW FO elements (45.9 m^2^) and six PF FO elements (42 m^2^) were used. The concentrations of FS and DS were 10 and 70 g/L, respectively.

Flow Rate Condition		SW FO			PF FO		
DS Flow (LPM)	FS Flow (LPM)	$\frac{Q_{feed,\;out}}{Q_{feed,\;in}}$	Theoretical $\frac{Q_{feed,\;out}}{Q_{feed,\;in}}$	$\frac{R_{exp}}{R_{max}}$	$\frac{Q_{feed,\;out}}{Q_{feed,\;in}}$	Theoretical $\frac{Q_{feed,\;out}}{Q_{feed,\;in}}$	$\frac{R_{exp}}{R_{max}}$
5	10	0.57	0.34	0.64	0.51	0.34	0.74
	15	0.63	0.40	0.59	0.62	0.41	0.63
	20	0.71	0.46	0.51	0.69	0.46	0.58
10	10	0.41	0.25	0.79	0.41	0.26	0.78
	15	0.55	0.30	0.63	0.54	0.30	0.65
	20	0.63	0.34	0.54	0.64	0.34	0.55
